# Non-isolated high gain DC–DC converter with ripple-free source current

**DOI:** 10.1038/s41598-024-51584-9

**Published:** 2024-01-10

**Authors:** A. S. Valarmathy, M. Prabhakar

**Affiliations:** 1 School of Electrical Engineering (SELECT), Vellore Institute of Technology, Chennai, 600127 India; 2 Centre for Smart Grid Technologies, School of Electrical Engineering (SELECT), Vellore Institute of Technology, Chennai, 600127 India

**Keywords:** Engineering, Energy science and technology, Renewable energy

## Abstract

In this paper, an interleaved DC–DC converter with high voltage gain capability is presented. The proposed converter is synthesized from a coupled-inductor (CI) based interleaved boost converter (IBC). For enhancing the voltage gain capability, voltage-lift capacitor, and diode-capacitor multiplier (DCM) cells are employed at the primary and secondary sides of the CIs. The proposed hybrid gain extension concept is practically validated using simulation and experimentation. A 185W prototype version of the proposed converter is switched at 50 kHz under laboratory conditions from a 18 V input to realize 380 V at the output port. The switches in the proposed converter operate at 0.5 duty ratio and experience a very low voltage stress of only 10.5% of the output voltage. Moreover, due to the interleaving mechanism, the input current ripple is just 11% of the total input current and the current rating of the switches is halved. Due to the adopted gain extension mechanism, the voltage stress on almost all the diodes is also significantly reduced. The swift dynamic response of the converter under closed-loop conditions is also practically demonstrated. Further, the beneficial features of the proposed converter are clearly validated by benchmarking its parameters with many state-of-the art converters which are available in literature.

## Introduction

The continuous increment in electrical energy demand and the simultaneous decline in the availability of fossil fuels has attracted engineers to tap renewable energy sources (RES) such as solar, wind and fuel cell^1,2^. Generally, photovoltaic (PV) panels yield low voltage at its output and need significant voltage transformation for connecting the load and other fruitful utilization purposes. An intermediate power electronic converter is generally adopted to boost the output from the PV panel^3,4^.

The classical boost converter (CBC) suffers from diode reverse-recovery, high voltage stress on the device and high-power loss especially when the switch is operated under extreme duty ratio (D > 0.8) values to meet the high voltage gain requirement. Therefore, it is customary to incorporate additional voltage gain extension circuits such as switched capacitors (SC), switched inductors (SI), voltage multiplier cells (VMCs) and diode-capacitor multiplier (DCM) cells within the CBC structure to achieve voltage gain values greater than 10^5–7^.

To achieve high voltage gain value from a compact structure, coupled inductors (CIs) are employed instead of discrete inductors in boost-derived DC–DC converters. In CI based converters, the converter’s voltage gain increases proportionately to the CIs’ turns-ratio value^8–10^. Incorporating additional voltage gain extension cells like VMCs and DCMs yields higher voltage gain values in CI based converters^11–14^.

The converters presented in^15,16^ utilize variations in the CIs like dual coupled inductors to achieve high voltage conversion ratio value. However, due to the leakage inductance of the CIs, the switches in these converters experience slightly higher voltage stress. The stored energy in the leakage inductance is suitably recycled through clamp circuits to reduce the voltage spike across the devices^17,18^.

The converters described in^19–21^ employ three winding arrangements of CI to achieve high voltage gain values. To balance the input current drawn from the source, various combinations of CIs like dual cross-coupled CIs are employed in^22^. However, CIs with multiple windings are rarely preferred due to the complexities in design, manufacturing, and the difficulties in controlling the leakage inductance of CIs.

For PV applications, smooth and ripple-free input current is best suited to implement maximum power point tracking (MPPT) algorithm efficiently. Generally, the input ripple current is minimized by employing a large energy storage inductor in boost-derived converters. However, large energy storage inductor increases the size and weight of the converter. Interleaving technique almost nullifies the input ripple current and is successfully employed to obtain a family of converters in^23^. The converters presented in^23^ yield high voltage gain values besides drawing ripple-free input current. The converters presented in^24,25,28^ use three and two CIs in an interleaved configuration. Their input current ripple is also negligible. To further enhance the voltage gain values, the turns-ratio of the CIs are adjusted and hybrid combinations of gain extension techniques like voltage lift technique, VMCs^26–30^ are adopted in conjunction with the interleaved arrangement. However, employing many turns in the CIs is likely to increase the leakage current and the consequent voltage spikes across the switches.

In^31^, a soft-switched multi-phase IBC is proposed for electric vehicles (EV) applications. The converter employs an auxiliary resonant circuit for achieving soft-switching behaviour. The converter described in^32^ employs a multi-phase interleaved buck-boost converter for DC–DC followed by DC-AC conversion system. The converter provides soft start-up and operates at near unity power factor values.

In this paper, a two-phase interleaved CI-based DC–DC converter with voltage lift capacitor and DCMs as gain extension mechanisms is presented. The manuscript is organized as follows: Section"Introduction" introduces the significance of the proposed converter synthesis while the power circuit is explained in section "Structure of proposed converter". The operating principle of proposed converter along with the characteristic waveforms is elaborated in section "Modes of operation". The expression for voltage gain and other key design expressions are derived and presented in section "Steady state analysis and design details" while the experimental results of proposed converter are discussed in section "Hardware results and discussion". In section "Performance Analysis and Comparison", the proposed converter is compared with some existing state-of-the art converters and the concluding remarks are presented in section "Conclusion".

## Structure of proposed converter

**Figure 1:**
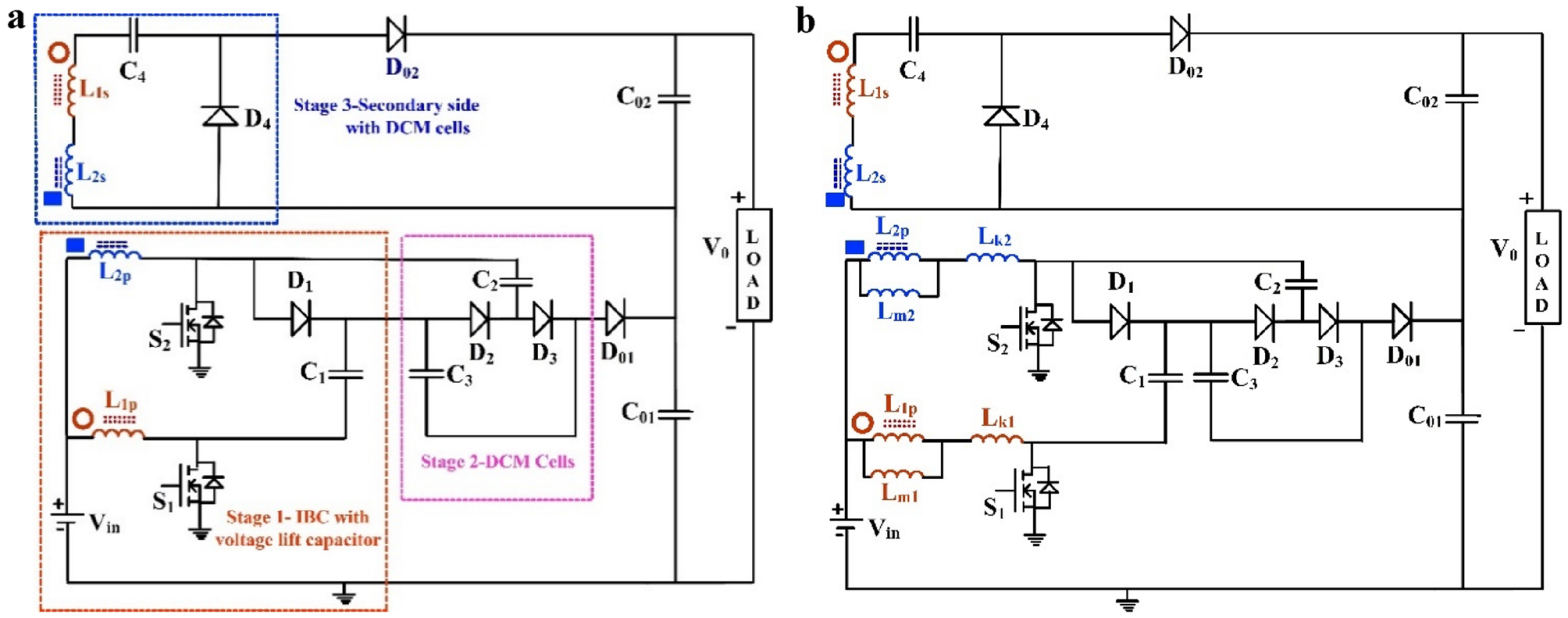
(**a**) Power circuit diagram of the proposed NI-HGIC. (**b**) Equivalent circuit of the proposed NI-HGIC.

The power circuit schematic of proposed high gain interleaved DC–DC converter derived from a fundamental two-phase IBC is portrayed in Fig. 1a. The proposed non-isolated high gain interleaved DC–DC converter (NI-HGIC) is synthesized from three stages. The interleaved structure formed by the two switches (*S*_*1*_ and *S*_*2*_), primary winding of the two CI's (*L*_*1p*_, *L*_*2p*_) along with voltage-lift capacitor (*C*_*1*_) is treated as Stage 1. Stage 2 of the proposed NI-HGIC consists of two DCM cells (*D*_*2*_*-C*_*2*_*, D*_*3*_*–C*_*3*_). The voltage obtained by cascading Stages 1 and 2 is coupled to the output capacitor *C*_*01*_. The secondary windings of the two CIs (*L*_*1s*_, *L*_*2s*_) along with the lone DCM pair (*D*_*4*_–*C*_*4*_) completes Stage 3 of the proposed NI-HGIC. The output obtained from the Stage 3 is connected to the output capacitor *C*_*02*_. The net voltage obtained from the proposed NI-HGIC is tapped by cascading *C*_*01*_ and *C*_*02*_. The CI is modelled as a combination of magnetizing and leakage inductors along with an ideal transformer with *1:n* turns ratio. The equivalent circuit is depicted in Fig. 1b. The operating principle of the converter is detailed in next section.

## Modes of operation

The operation of the proposed NI-HGIC is explained using two distinct modes in one switching cycle by assuming that all the circuit components are ideal and the converter operates in continuous conduction mode (CCM). These assumptions are later relaxed by including the non-idealities when obtaining the loss distribution profile of the converter. Further, since the proposed NI-HGIC is intended to be employed in renewable energy application, CCM is ensured by properly designing the primary inductance value.

### ***Mode 1 (t***_***o***_***-t***_***1***_***)***

Mode 1 commences at time *t* = *t*_*o*_ when *S*_*1*_ is turned ON and *S*_*2*_ is turned OFF. As *S*_*1*_ is ON, the magnetizing inductor *L*_*m1*_ and the leakage inductor *L*_*k1*_ starts to charge linearly towards *V*_*in*_ through *S*_*1*_. During this energy storage process of CI_1_, *D*_*2*_ is reversed biased due to the polarity of voltage across* C*_*2*_ and *C*_*3*_. Since *S*_*2*_ is OFF, the stored energy in magnetizing inductor *L*_*m2*_, leakage inductor *L*_*k2*_ and *C*_*2*_ forward biases *D*_*3*_ and is transferred to *C*_*3*_; it charges through *S*_*1*_. Depending on the states of *L*_*1p*_ and *L*_*2p*_, the secondary windings *L*_*1s*_ and *L*_*2s*_ discharge and charge respectively at the secondary side. The energy stored in the secondary winding and *C*_*4*_ is transferred to *C*_*02*_ through *D*_*02.*_ Mode 1 ends when the current through* L*_*2s*_ just reaches zero. The primary and secondary current of CIs are given by (1)–(3).

1 $$i_{{L}_{{{1p} }}} (t) = i_{{S}_{{{{1}} }}} (t) - i_{{C}_{{{{1}} }}} (t)$$

2 $$i_{{L}_{{{{2{\text{p}}}} }}} (t) = \frac{{V_{in} - v_{{C_{1} }} (t)}}{{L_{{2{\text{p}}}} }}t$$

3 $$i_{{L}_{{{2s} }}} (t) = i_{{D}_{{{4} }}} (t) = \frac{1}{n}i_{{L}_{{{{2{\text{p}}}} }}} (t)$$

### ***Mode 2 (t***_***1***_***-t***_***2***_***)***

During Mode 2, since *S*_*1*_ is ON, the energy stored in *L*_*m1*_ continues to rise while the magnetizing inductor *L*_*m2*_ is completely transferred to *C*_*2*_ and *C*_*3*_*.* At the secondary side, the energy stored in *L*_*1s*_ is transferred to *C*_*02*_. Since *L*_*2s*_ charges, its current starts to raise while the current through *L*_*1s*_ becomes negative. Mode 2 ends at *t*_*2*_ when *S*_*2*_ is ready to be turned ON.

### ***Mode 3 (t***_***2***_***-t***_***3***_***)***

During Mode 3, the anti-body diode of *S*_*2*_ is forward-biased and begins to conduct. Resultantly, a small negative current is realized through *S*_*2*_. The energy stored in *L*_*k1*_ reaches its peak value while the current through *L*_*k2*_ reaches zero and turns-OFF the anti-body diode of *S*_*2*_. Thus, the energy storage and transfer processes of *L*_*1p*_ and *L*_*2p*_ respectively ends at time *t* = *t*_*3*_.

### ***Mode 4 (t***_***3***_***-t***_***4***_***)***

Mode 3 commences at *t* = *t*_*3*_, when switch *S*_*2*_ is turned ON and *S*_*1*_ is turned OFF. As *S*_*2*_ is ON, magnetizing inductor *L*_*m2*_ operates in the energy storage mode and starts to charge linearly towards *V*_*in*_. Since *S*_*1*_ is OFF, the energy stored in magnetizing inductor *L*_*m1*_ and leakage inductor* L*_*k1*_ is transferred to *C*_*2*_ through *D*_*2*_. The polarity of voltage across *C*_*1*_ reverse biases *D*_*1*_. At the secondary side, *L*_*2s*_ operates in energy discharge interval while *L*_*1s*_ stores energy. The net energy stored in the secondary windings is transferred to *C*_*4*_ through *D*_*4*_ while *D*_*02*_ remains in reverse-biased condition. Mode 4 ends when current through *L*_*1s*_ reaches zero. The currents through the primary and secondary windings of the CIs during Mode 4 are given by (4)–(6).

4 $$i_{{L}_{{{{1{\text{p}}}} }}} (t) = \frac{{V_{in} + v_{{C_{1} }} (t) - v_{{C_{2} }} (t)}}{{L_{{1{\text{p}}}} }}t$$

5 $$i_{{L}_{{{{2{\text{p}}}} }}} (t) = i_{{S}_{{{2} }}} (t) - i_{{C}_{{{2} }}} (t)$$

6 $$i_{{L}_{{{1s} }}} (t) = \frac{1}{n}i_{{L}_{{{{{\text{1p}}}} }}} (t)$$

### ***Mode 5 (t***_***4***_***-t***_***5***_***)***

During Mode 5, the energy stored in *L*_*m2*_ continues to rise while the magnetizing inductor *L*_*m1*_ continues to transfer its stored energy to *C*_*1*,_* C*_*3*_ and *C*_*01*_*.* At the secondary side, the DCM capacitor *C*_*4*_ stores energy while the output capacitor *C*_*02*_ transfers its stored energy to the load. Mode 5 ends when S_1_ is ready to be turned ON again.

### ***Mode 6 (t***_***5***_***-t***_***6***_***)***

During Mode 6, *S*_*2*_ remains in the ON state. The anti-body diode of *S*_*1*_ is forward-biased due to the potential difference between its anode and cathode terminals. Hence, current through S_1_ starts flowing from the ground terminal towards *C*_*1*_ and results in a negative current through *S*_*1*_. The current through *L*_*k1*_ reaches zero and the anti-body diode of *S*_*1*_ turns OFF at *t* = *t*_*6*_, thus marking the end of one switching cycle.

**Figure 2:**
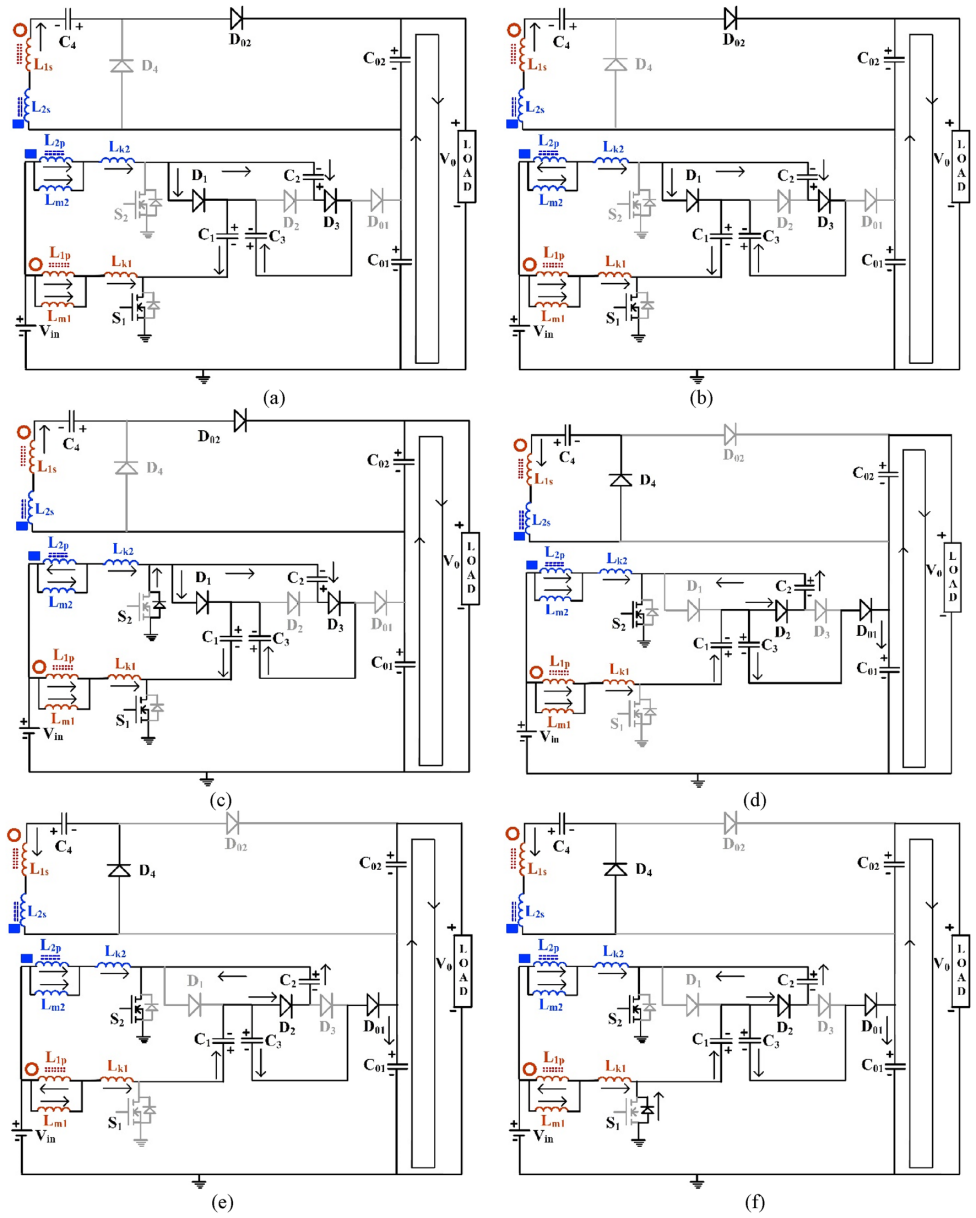
Diagrams of the conducting devices and current paths during (**a**) Mode 1, (**b**) Mode 2, (**c**) Mode 3, (**d**) Mode 4, (e) Mode 5, and (**f**) Mode 6.

**Figure 3:**
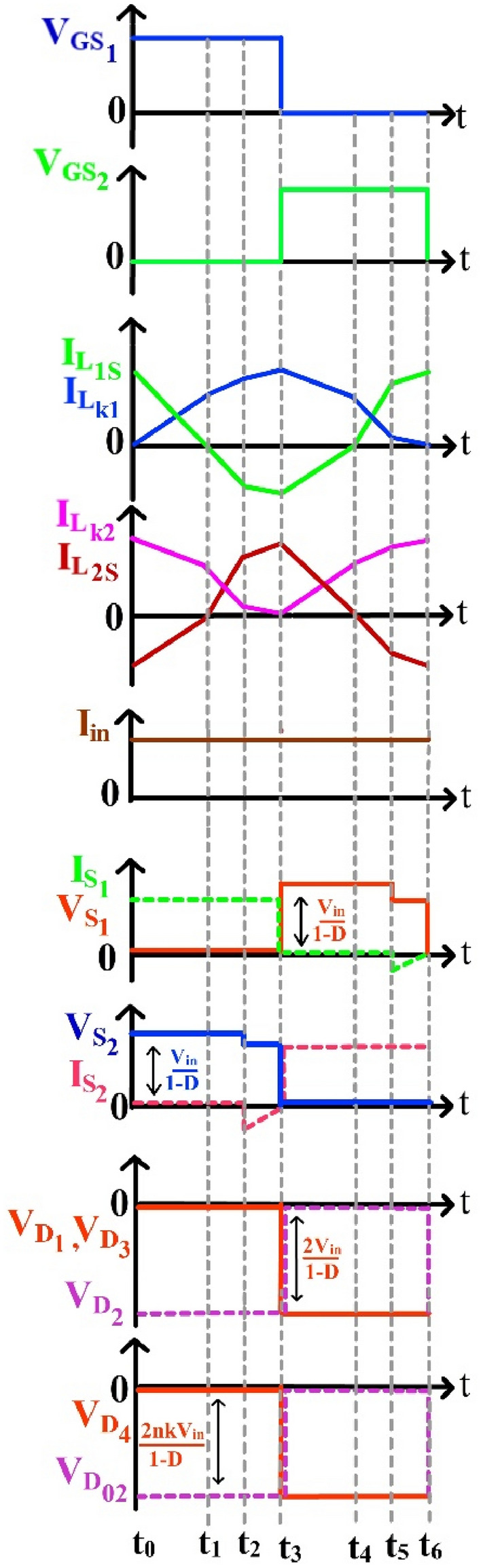
Characteristic waveforms of the proposed NI-HGIC.

The diagrams of the conducting devices and current paths during Modes 1 to 6 are depicted through Fig. 2a–e respectively. The characteristic waveforms of the key parameters of the proposed NI-HGIC are portrayed in Fig. 3 for one switching cycle. In the subsequent section, the design equations for the converter are derived.

## Steady state analysis and design details

### Voltage Gain

The voltage gain expression of the proposed NI-HGIC is derived from the volt-second balance equations. The overall voltage gain of the converter is obtained by deducing the voltage gain contributed by Stage 1 and Stage 2. The charging of primary inductors *L*_*1p*_ and *L*_*2p*_ towards V_in_ occurs when the respective switches *S*_*1*_ and *S*_*2*_ are ON. The discharge of the inductors occurs when switches *S*_*1*_ and *S*_*2*_ are OFF. Thus, the voltage in the inductors in ON state and OFF states are given by (7)–(11).

7 $$v_{{L_{1} {\text{p}}(ON)}} = V_{in}$$

8 $$v_{{L_{1} {\text{p}}\,(OFF)}} = V_{in} - V_{{C_{1} }}$$

9 $$v_{{L2{\text{p}}(ON)}} = V_{in}$$

10 $$v_{{L2{\text{p}}(OFF)}} + V_{in} + V_{{C_{3} }} = V_{{C_{01} }}$$

Capacitor* C*_*1*_ is voltage lift capacitor and its voltage is given by (11).

11 $$V_{{C_{1} }} = \frac{2}{1 - D}V_{in}$$

where *D* is the duty ratio of *S*_*1*_ and *S*_*2*_.

Considering the voltage gain contributed by the two DCMs employed in Stage-2 of converter, the net voltage gain contributed by Stage-1 and Stage-2 is impressed across the output capacitor *C*_*01*_ and expressed as (12).

12 $$V_{{C_{01} }} = \frac{4}{1 - D}V_{in}$$

Since *C*_*02*_ is located at the secondary side of the CIs, the voltage developed across it is given by (13).

13 $$V_{{C_{02} }} = \frac{2nk}{{1 - D}}V_{in}$$

where *n* is the turns ratio of coupled inductor, *k* represents coupling coefficient.

The net output voltage obtainable from the proposed NI-HGIC is derived by summing up the voltages obtained across its output capacitors *C*_*01*_ and *C*_*02*_ and given by (14).

14 $$V_{0} = V_{{C_{01} }} + V_{{C_{02} }} = \frac{4}{1 - D}V_{in} + \frac{2nk}{{1 - D}}V_{in}$$

The generalized voltage gain expression with ‘*N*’ number of DCM cells is given by

15 $$\frac{{V_{0} }}{Vin} = M = \underbrace {{\frac{2}{1 - D} + }}_{\begin{subarray}{l} {\text{Stage - 1 IBC}} \\ {\text{ with C}}_{{{\text{Lift}}}} \end{subarray} } \, \underbrace {{ \, \frac{N}{1 - D} + }}_{\begin{subarray}{l} {\text{ Stage - 2}} \\ {\text{DCM cells}} \end{subarray} } \, \underbrace {{\frac{2nk}{{1 - D}}}}_{\begin{subarray}{l} {\text{Stage - 3 Secondary}} \\ {\text{ side with DCMs}} \end{subarray} }$$

### Voltage stress across switch

The switches in proposed NI-HGIC are located at the same position as that of the switches in CBC. Hence, their voltage stress is given by (16).

16 $$V_{{S_{1} }} = V_{{S_{2} }} = \frac{{V_{in} }}{1 - D}$$

As the voltage stress in switches *S*_*1*_ and *S*_*2*_ are low, switches with low voltage rating are sufficient to achieve a high voltage gain value. In terms of output voltage *V*_*0*_, (15) is rearranged and expressed as depicted in (17).

17 $$V_{{S_{1} }} = V_{{S_{2} }} = \frac{{V_{0} }}{2 + N + 2nk}$$

### Voltage stress on the diodes

Voltage rating of diodes is determined by the reverse potential difference between anode and cathode terminals. When *D*_*1*_ is OFF, its anode terminal is grounded through *S*_*2*_ and its cathode is maintained at the potential of *C*_*1*_. Similarly, diodes in DCM cells experience voltage stress based on the potential at their terminals. The voltage stress experienced by all diodes in DCM cells is given by (18).

18 $$V_{{{D_{1} }}_{{ - D_{3} }}} = \frac{2}{1 - D}V_{in} = = \frac{{2V_{0} }}{2 + N + 2nk}$$

The voltage stress on the output diode *D*_*01*_ is given by (19).

19 $$V_{{D_{01} }} = \frac{2 + N}{{1 - D}}V_{in} = \frac{{\left( {2 + N} \right)V_{0} }}{2 + N + 2nk}$$

The voltage stress on *D*_*4*_ and *D*_*02*_ is given by (20).

20 $$V_{{D_{4} }} = V_{{D_{02} }} = \frac{2nk}{{1 - D}}V_{in} = \frac{{2nkV_{0} }}{2 + N + 2nk}$$

### Current stress on semiconductor devices

When *S*_*1*_ is ON, *S*_*2*_ is maintained in the OFF state. The current flowing through *S*_*1*_ and *S*_*2*_ is sum of currents through *L*_*1p*_ and *L*_*1s*_ and expressed by (21) and (22).

21 $$I_{{S_{1} }} = I_{{L_{{1{\text{p}}}} }} + I_{{L_{1s} }}$$

22 $$I_{{S_{2} }} = I_{{L_{{2{\text{p}}}} }} + I_{{L_{2s} }}$$

In terms of the input current, due to the interleaved structure, the input current is shared between the two phases and is expressed by (23).

23 $$I_{{S_{2} }} = I_{{S_{1} }} = \frac{{I_{in} }}{2}$$

Current stress of *D*_*1*_ is obtained by considering the voltage gain at its terminals. Thus, current rating of the diodes *D*_*1*_-*D*_*3*_ is given by (24) and (25).

24 $$I_{{D_{1} }} = \left( {1 - D} \right)I_{in}$$

25 $$I_{{D_{2} }} = I_{{D_{3} }} = \frac{(1 - D)}{2}I_{in}$$

Diode *D*_*4*_ is in the Stage 3 which is formed by the secondary windings of the CIs. Hence, its current rating is given by (26).

26 $$I_{{D_{4} }} = \frac{(1 - D)}{{2nk}}I_{in}$$

Diode *D*_*01*_ is connected at the output of Stage 1 and the output current flows through *D*_*01*_, its current rating is (27).

27 $$I_{{D_{01} }} = \frac{(1 - D)}{{(1 + N)}}I_{in}$$

Likewise, due to the location of *D*_*02*_ in Stage 2, its current stress is given by (28).

28 $$I_{{D_{02} }} = \frac{(1 - D)}{{2nk}}I_{in}$$

### Design expressions for passive component ratings

The primary inductance values of the CIs are influenced by ripple current through the individual inductors, voltage at input, switching frequency and duty ratio of switches. Hence, the design expression is represented by (29).

29 $$L_{py} = L_{{1{\text{p}}}} = L_{{2{\text{p}}}} = \frac{{V_{in} \,D}}{{2f\Delta i_{{L_{py} }} }}$$

The value of the secondary side inductances is determined using the CIs’ turns-ratio and is expressed using (30).

30 $$L_{sy} = n^{2} L_{py}$$

The value of the capacitances is impacted by their energy storage capability and voltage ripple impressed across them. From basic principles, the expression for computing the capacitance values is expressed using (31).

31 $$C_{x} = \frac{{\,I_{x} \,D}}{{f\Delta v_{{C_{x} }} }}$$

where *x* represents 1, 2, 3 and 4. The capacitance values of the output capacitors are determined from (32).

32 $$C_{0} = C_{01} = C_{02} = \frac{{\,I_{0} \,D}}{{f\Delta v_{{C_{0} }} }}$$

## Hardware results and discussion

Experiments are carried out on a laboratory prototype version of the proposed NI-HGIC with the specifications mentioned in Table 1. The components employed to build and test the prototype converter are also mentioned.

**Table 1:** Specifications of the proposed converter and the components used in the proposed NI-HGIC.

Parameter	Value	Circuit element	Part no. (ratings)
Input voltage (*V*_*in*_)	18 V	Switches *S*_*1*_, *S*_*2*_	FQP33N10 (100 V, 33A, 52mΩ)
Output voltage (*V*_*o*_)	380 V	Diodes *D*_*1,*_* D*_*2*_, *D*_*3*_	HTE5L100 (100 V, 5A, 0.52 V)
Output power (*P*_*o*_)	185W	Diodes *D*_*4*_, *D*_*01*_, *D*_*02*_	MUR460 (400 V, 4A, 1.05 V)
Switching frequency (*f*)	50 kHz	Inductances *L*_*1p*_ / *L*_*1s*_ and *L*_*2p*_ / *L*_*2s*_	80μH,10A / 1200μH, 2A
Duty ratio (*D*)	0.5	Capacitors *C*_*1*_-*C*_*4*_ *C*_*01*,_ *C*_*02*_	4.7μF/100 V (Polypropylene) 47μF/250 V, 47μF/400 V (Electrolytic)

**Figure 4:**
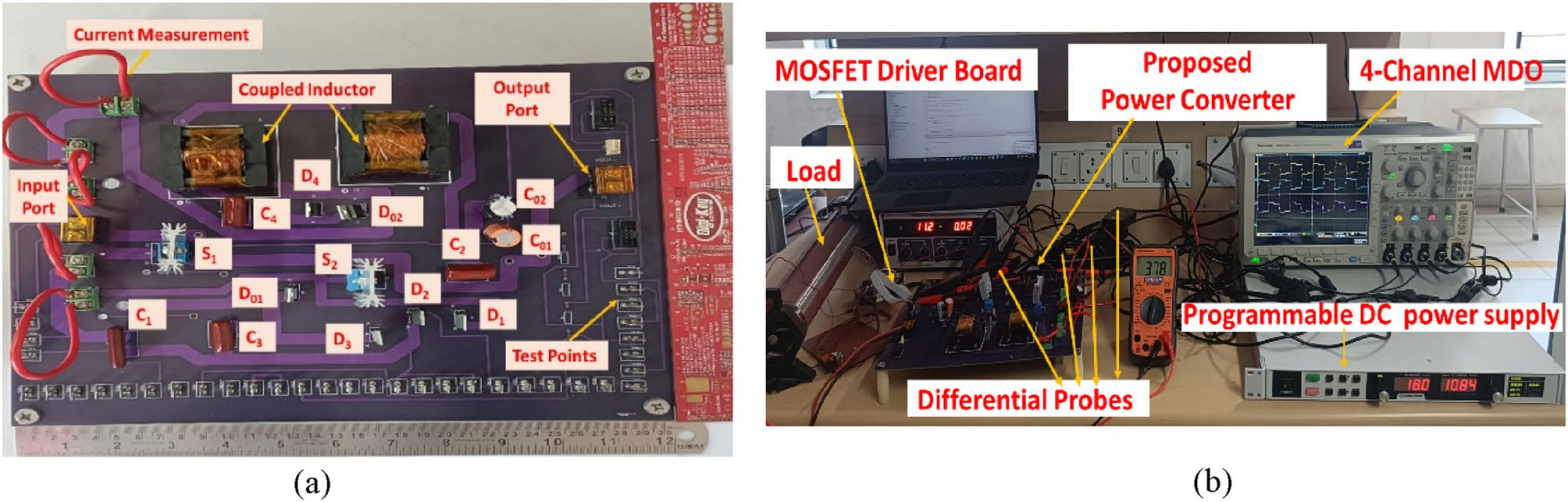
Photograph of (**a**) the prototype version of the proposed NI-HGIC. (**b**) The experimental set up used to test the NI-HGIC.

Figure 4a depicts the photograph of proposed NI-HGIC and its experimental setup photograph is depicted in Fig. 4b. The gate pulses for the switches are generated by suitably programming a STM32F411RE microcontroller. The gate pulses are then applied to a MOSFET driver IRF25600 before interfacing them with the power circuit. Tektronix mixed domain oscilloscope (MDO4014C) along with standard accessories like differential high voltage probes (P5200A) and current probes (A622) are used to capture the experimental waveforms from proposed NI-HGIC.

**Figure 5:**
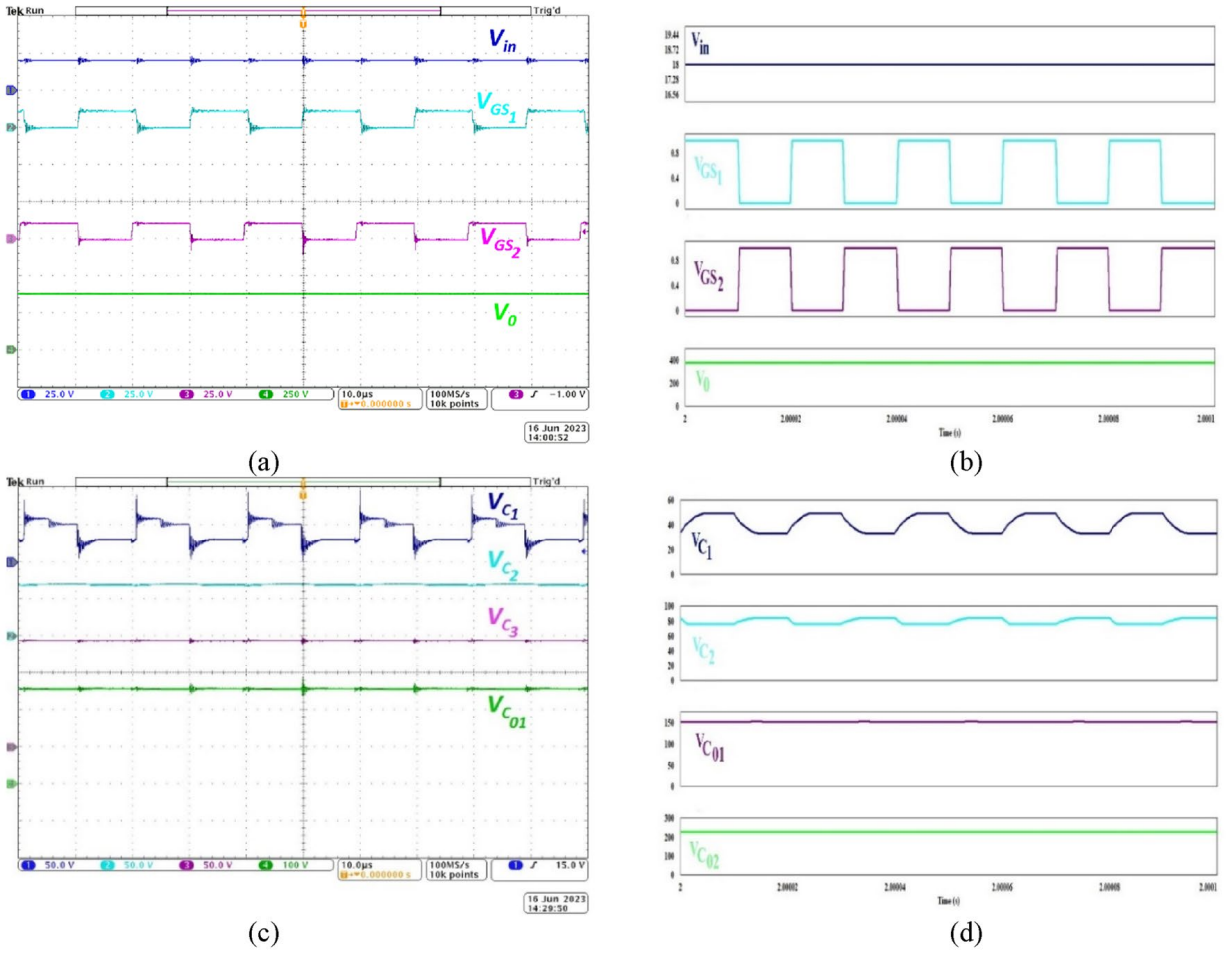
Waveforms exhibiting voltage gain capacity during (**a**) experimentation, (**b**) simulation and voltage gain enhancement concept during (**c**) experimentation and (**d**) simulation.

Figure 5a,b respectively depict the experimental and simulated waveforms for the gate pulses (CH1, CH2), input voltage (CH3) and voltage measured across the load terminals (CH4). The proposed NI-HGIC employs an interleaved structure. The gate pulses to *S*_*1*_ and *S*_*2*_ are phase shifted by 180° with a duty ratio of 0.5 and 50 kHz switching frequency. When 18 V is supplied as the input, the converter produces 380 V at the output. This validates the practical voltage conversion ratio of 21.11. Thus, the proposed hybrid gain extension technique combining CIs, voltage lift capacitors and DCMs employed in the proposed NI-HGIC is validated.

The practical voltage waveforms across *C*_*1*_*, C*_*2*_*, C*_*3*_*, C*_*01*_ are captured and presented in Fig. 5c through the channels CH1, CH2, CH3 and CH4 respectively. Since the voltage lift technique is used, the voltage that is obtained across *C*_*1*_, between the top plate and ground is dependent upon the states of *S*_*1*_ and *S*_*2*_. The bottom plate of *C*_*1*_ is grounded when *S*_*1*_ is ON held at a potential which is equivalent to that of CBC when *S*_*1*_ is OFF. Therefore, its voltage swings periodically as depicted through the CH1 waveform. The voltage across the DCM capacitors *C*_*2*_ and *C*_*3*_ (CH2, CH3 respectively) clearly validates the voltage gain contributed by DCM cells. By observing the voltage across *C*_*01*_ (CH4) the net contribution of Stages 1 and 2 is also validated. Thus, the circuit synthesis and its proper operation is practically demonstrated and verified. Figure 5d portrays the simulated waveforms of the same parameters as in Fig. 5c.

**Figure 6:**
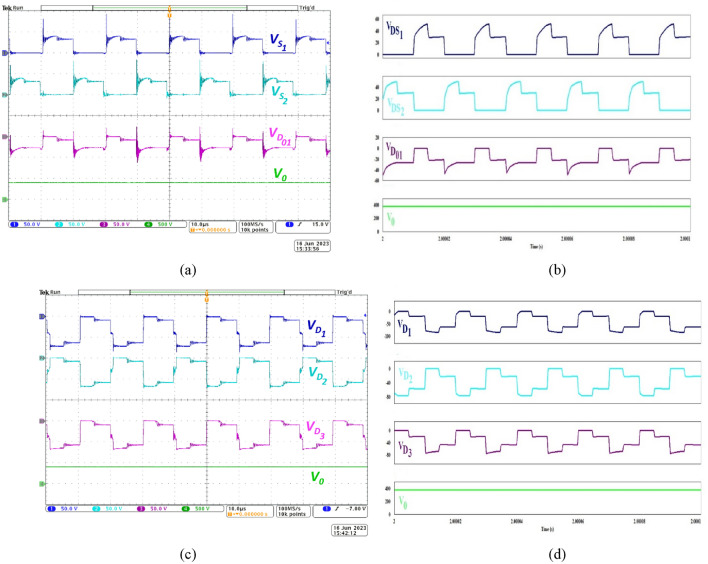
Waveforms demonstrating the voltage stress on *S*_*1*_ and *S*_*2*_ with respect to output voltage during (**a**) experimentation and (**b**) simulation, (**c**) correlated operation of *D*_*1*_, *D*_*2*_, *D*_*3*_ and *V*_*0*_ profiles while experimenting and (**d**) simulating.

Figure 6a,b respectively depict the practical and simulated values of voltage stress experienced by the *S*_*1*_, *S*_*2*_ and the output diode *D*_*01*_. The switches are employed at the two legs of the IBC structure and are operated with 180° phase-shift. Hence, their complementary operation is validated. Additionally, when *S*_*1*_ is ON, the passive elements located in Stages 1 and 2 store energy and the output diode *D*_*01*_ remains in reverse-biased state. The correlation between the switches and *D*_*01*_ is also verified from Fig. 6a. Interestingly, in the gain extension mechanism adopted in the proposed NI-HGIC, the switches are judiciously located closer to the input port. Consequently, *S*_*1*_ and *S*_*2*_ are subjected to very low voltage stress value which is only 10.5% of output voltage (CH4). The voltage spikes observed in the waveforms are caused by the leakage inductance of CIs. The voltage spikes in the waveforms are within the safe limits. The voltage across output diode *D*_*01*_ (CH3) clearly depicts the complementary operation of* S*_*1*_ and *D*_*01*_ as expected. The slight increase in voltage stress magnitude of* D*_*01*_ is mainly due to its proximity to the output port and its voltage stress magnitude matches with the value calculated using (18).

Figure 6c depicts the proper operation of diodes *D*_*1*_*, D*_*2,*_* D*_*3*_ and their voltage stress levels compared to *V*_*0*_ under practical conditions while the simulation results are portrayed in Fig. 6d. The diodes operate in a complementary manner as elaborated during the circuit operation and is illustrated through the practical voltage waveforms presented in CH1, CH2 and CH3 respectively. The voltage stress magnitude of *D*_*1*_, *D*_*2*_ and *D*_*3*_ is 72 V and is consistent with the value predicted using (17). Compared with the output voltage, the voltage stress level works out to 18.95% of *V*_*0*_. Since the DCM cells are adopted, each diode in the cell is subjected to a lower voltage stress magnitude as discussed theoretically and verified practically.

**Figure 7:**
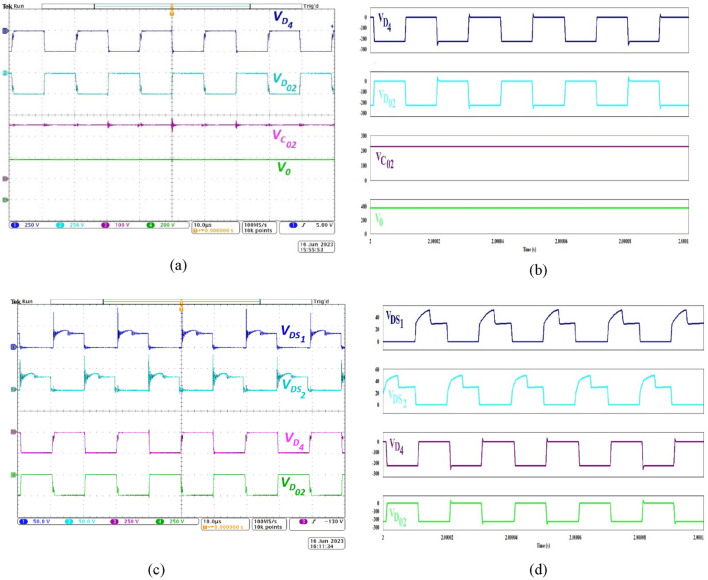
Waveforms demonstrating the correlated operation of *D*_*4*_, *D*_*02*_, *C*_*02*_, *V*_*0*_ during (**a**) experimentation, (**b**) simulation, (**c**) experimental waveforms of voltage stress on *S*_*1*_ and *S*_*2*_ in accordance with* D*_*4*_ and *D*_*02*_ and (**d**) simulated waveforms.

Figure 7a demonstrates the complementary operation of *D*_*4*_, *D*_*02*_ (CH1, CH2), voltage across the secondary-side capacitor *C*_*02*_ (CH3) and the output voltage (CH4) during experimentation. Expectedly, the voltage developed across *C*_*02*_ of Stage 3 in the proposed NI-HGIC is validated by (13). Since *D*_*02*_ is located at the secondary side of the CIs, its voltage stress magnitude is very close to the voltage impressed across *C*_*02*_. Under simulated condition, the proposed converter exhibits similar behaviour as observed from Fig. 7b.

Figure 7c,d are used to validate the correlated operation of the switches (*S*_*1*_-CH1, *S*_*2*_-CH2) which are located at Stage 1 and the diodes located in Stage 3 (*D*_*4*_-CH3, *D*_*02*_-CH4) during practical and simulated conditions respectively. The practical waveforms prove that when *S*_*1*_ is ON, *D*_*4*_ is reverse-biased and *D*_*02*_ conducts. Thus, the diodes employed at the secondary-side of the CIs contribute to the voltage gain extension through Stage 3 of the proposed NI- HGIC. To summarize, the two switches and all the diodes employed in the NI-HGIC operate as expected and their voltage stress magnitudes are experimentally verified.

**Figure 8:**
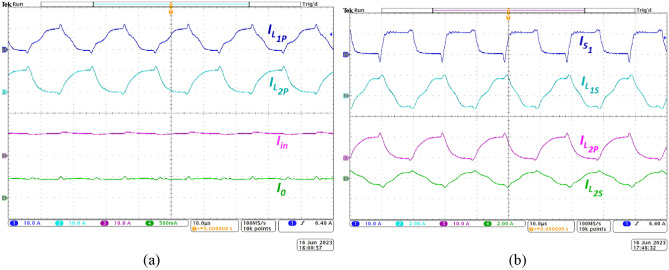
Experimental waveforms of current through (**a**) *L*_*1P*_*, L*_*2P*_, the input (*I*_*in*_) and the output (*I*_*o*_), (**b**) switch *S*_*1*_, *L*_*1s*_, *L*_*2P*_, and *L*_*2s*_*.*

The experimental waveforms of the primary inductor currents (CH1, CH2) along with the input and output currents (CH3, CH4) are portrayed in Fig. 8a. CH1 and CH2 reveal the complementary charging and discharging profiles of *L*_*1p*_ and *L*_*2p*_ respectively. The interleaved arrangement employed in the NI-HGIC results in sharing of the input current by the primary windings of CI. Experimental waveforms indicate that the proposed NI-HGIC draws 10.8A from the source under full-load condition. Further, due to the operation of switches at *D* = 0.5 with 180° phase-shift, the input current is free from ripples as observed from the practical waveforms (CH3). In fact, though the current through the individual inductors contain ripples, they are nullified due to the interleaved operation and the net input current is almost ripple-free. Due to the manufacturing imperfections, small current spikes are observed at the switching instants. Hence, the ripple content is calculated to be 11.11% of the total input current magnitude. Further, based on the voltage gain achieved, the output current magnitude (CH4) is observed and to be 0.48A. Thus, the proposed NI-HGIC delivers 185W power to the load at an output voltage of 380 V.

Figure 8b shows experimental waveforms of current through *S*_*1*_ (CH1), secondary winding *L*_*1s*_ (CH2), primary winding *L*_*2p*_ (CH3) and secondary winding *L*_*2s*_ (CH4). As explained in Section "Modes of operation", currents through the secondary windings *L*_*1s*_ and *L*_*2s*_ exhibit an alternating (AC) behaviour due to their charging and discharging intervals. Their magnitudes are also on expected lines. Thus, the correlated operation of the switch current and the inductor currents is experimentally verified.

**Figure 9:**
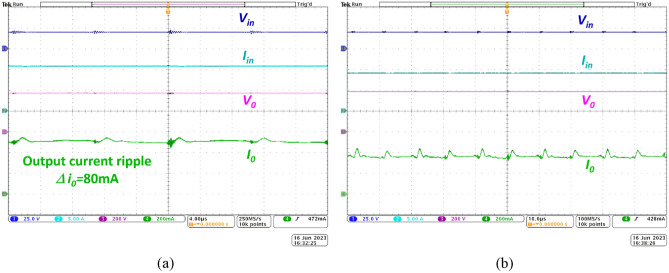
Practical waveforms of the proposed NI-HGIC to obtain efficiency (**a**) at full-load condition (185W) and (**b**) 150W.

The practical efficiency of the prototype NI-HGIC under full-load condition is extracted from waveforms depicted in Fig. 9a. Based on the values of the voltages and currents captured at the input and output terminals, the prototype NI-HGIC delivers 185W at 94.8% efficiency. Since the semiconductor devices are subjected to low voltage levels, their ratings are reduced mainly due to the adopted gain extension technique. At 150W power level, the proposed NI-HGIC delivers power to the load at 390 V as illustrated in Fig. 9b. Since the load on the converter is slightly reduced, the output voltage increases marginally and the efficiency is about 92%.

**Figure 10:**
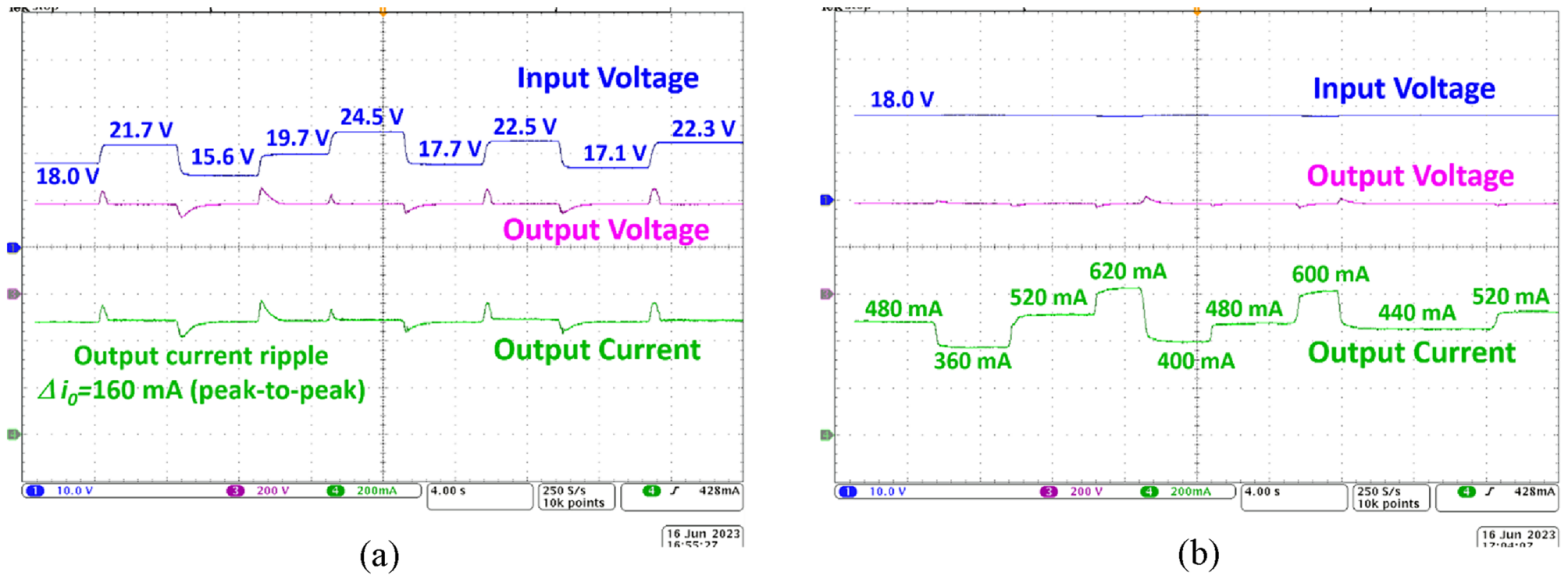
Dynamic response of the proposed NI-HGIC under closed-loop condition when (**a**) line voltage (CH1) varies and (**b**) load current (CH3) undergoes a step variation.

To regulate the output voltage obtained from the proposed NI-HGIC when the input voltage and/or the load current undergoes step variations, digital proportional-integral (PI) based closed-loop is implemented. The STM32F411RE microcontroller is suitably programmed to fetch the actual *V*_*0*_ value from the in-built analog-to-digital converter (ADC), compare it with the desired value (380 V) and generate the gate pulses to *S*_*1*_ and *S*_*2*_ using the timer module. Figure 10a depicts the dynamic response of the proposed NI-HGIC when the input voltage undergoes step variations. The output voltage obtained from the proposed NI-HGIC settles down quickly to the desired value of 380 V when the input voltage variation ranges from 15.6 V to 24.5 V. In absolute magnitude terms, the input voltage is variation is 8.9 V. Considering the nominal input voltage of 18 V, the experimental result proves the effectiveness of the closed-loop mechanism.

In Fig. 10b, the load regulation profile of the proposed NI-HGIC is depicted. Under full-load condition (185W at 380 V), the nominal load current value is 486 mA. When the load on the proposed NI-HGIC is varied from 360 to 620 mA in a stepped manner, the output voltage profile undergoes overshoots and undershoots depending on the light or heavy load conditions respectively. Nevertheless, the output voltage is restored back to its nominal 380 V due to the implemented closed-loop control technique. Importantly, the overshoot and undershoot values of the output voltage are within acceptable limits. Thus, the converter is expected to be suitable for a practical DC microgrid application.

**Figure 11:**
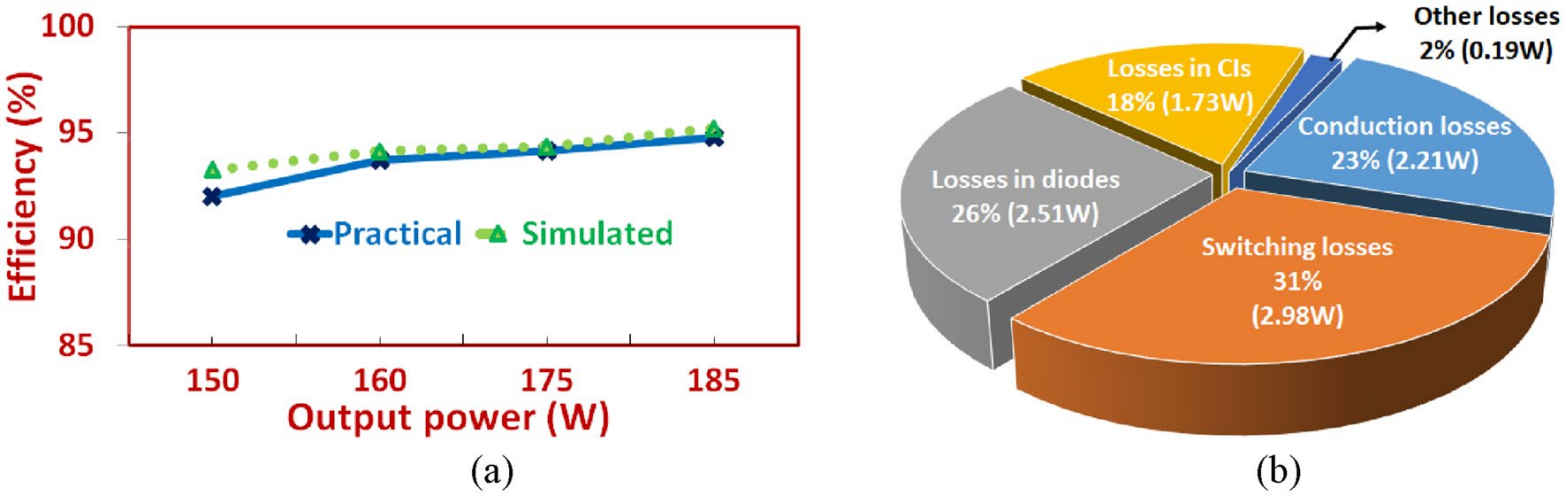
(**a**) Simulated and practical efficiency curves of proposed NI-HGIC, (**b**) Pie-chart to demonstrate various losses occurring in the proposed NI-HGIC.

The efficiency curve of the converter under various load conditions during simulation and experimentation is demonstrated through Fig. 11a. The practical values match closely with the simulated values. To understand and appreciate the various losses that occur in the proposed NI-HGIC, standard expressions presented in^25^ are used. The losses that occur across the parasitic elements of the passive elements and the semiconductor devices are calculated and represented as a pie-chart in Fig. 11b. Due to the use of low voltage rated semiconductor devices, their conduction losses are reduced.

## Performance analysis and comparison

The proposed NI-HGIC is compared with two converter categories viz., single and two switch based converters. Table 2 provides a glimpse of the comparison between the proposed NI-HGIC and some single-switch based converter versions. All the single-switch converters considered for comparison yield high voltage gain values ranging from 13.6 to 19. Despite employing a lone switch and lesser number of components than the NI-HGIC, their power handling levels are also reasonably good. Nevertheless, the proposed NI-HGIC outshines the compared converters mainly in the following attributes: (i) higher *M/TCC* value, (ii) lowest voltage stress on the two switches and (iii) lowest input current ripple. Since only a lone switch is employed, all the converters compared in Table 2 rely on the inductance value to limit the input current ripple. Consequently, the converter becomes bulky due to the necessity to deploy a higher inductance value. Despite employing smaller inductance values, the proposed NI-HGIC draws near ripple-free input ripple due to the interleaving mechanism adopted.

**Table 2:** Comparison of the proposed NI-HGIC with some high gain single-switch converters.

Attributes	High gain single-switch-based converters presented in references							Proposed NI-HGIC
	^7^	^11^	^12^	^13^	^17^	^18^	^20^	
Input voltage (*V*_*in*_)	11	24	24	50	28	12	20	18
Output voltage (*V*_*0*_)	180	354	361	400	382	200	380	380
Output power (*P*_*0*_)	200	416	260	200	226	120	300	185
Voltage gain (*M*)	16.4	14.75	15.04	16	13.6	16.67	19	21.1
Duty ratio (*D*)	0.65	0.6	0.6	0.5	0.51	0.6	0.62	0.5
Total component count (*TCC*)	12	11	15	14	14	12	18	16
*M/TCC*	1.36	1.34	1	1.14	0.97	1.38	1.06	1.32
Input ripple current Δ*i*_*in*_ (% of *I*_*in*_)	13	17	15.6	-	18.8	15	14	11.11
Voltage stress on the switch	$\frac{{V_{0} }}{{3 + n\left( {1 + D} \right)}}$	$\frac{{V_{in} }}{{\left( {1 - D} \right)^{2} }}$	S[12]	$\frac{{V_{0} }}{3 + 2n + nD}$	$\frac{{V_{0} }}{2n + 3}$	$\frac{{V_{0} }}{{\left( {3 - D} \right)N + 2}}$	$\frac{{V_{0} }}{{N_{sp} + 2}}$	$\frac{{V_{0} }}{2 + N + 2nk}$
Switch stress (% of *V*_*0*_)	15.80	42	17	20	14.28	15	12.5	10.50

In order to obtain a fair and deeper understanding on the superior features of the proposed NI-HGIC, some two-switch, CI based converters are compared and presented in Table 3. An in-depth analysis is elaborated in the subsequent sub-sections to appreciate the beneficial characteristics of the proposed NI-HGIC.

**Table 3:** Comparison of some state-of-the art two-switch-based high gain converters with the proposed NI-HGIC.

Attributes	State-of-the art two-switch CI-based converters presented in references				Proposed NI-HGIC
	^6^	^10^	^27^	^28^	
Input voltage (*V*_*in*)_	25	11	25	27	18
Output voltage (*V*_*0*_)	380	237	400	400	380
Output power (*W*)	100	150	200	600	185
Voltage gain (*M*)	15.2	21.5	16	15	21.1
Duty ratio (*D*)	0.78	0.75	0.6	0.61	0.5
*TCC*	11	12	13	11	16
*M/TCC*	1.382	1.8	1.2	1.35	1.32
Δ*i*_*in*_ (% of *I*_*in*_)	47	21	11	18.75	11.11
Voltage stress on the switch	$\frac{{\left( {M + 2} \right)V_{0} }}{4M}$	$\frac{{V_{0} }}{{3 + 2N_{b} + N_{a} }}$	$\frac{{V_{in} }}{{\left( {1 - D} \right)}}$	$\frac{{V_{0} }}{{N\left( {n + 1} \right) + 2}}$	$\frac{{V_{0} }}{2 + N + 2nk}$
Switch voltage stress (% of *V*_*0*_)	Min. 21 Max. 28.90	Min. 10.9 Max. 15.2	Min. 15.25 Max. 18.25	19 (both the switches)	10.50 (both the switches)
Maximum diode voltage stress	$\frac{{V_{0} }}{2}$	$\frac{{\left( {2 + N_{a} + N_{b} } \right)V_{0} }}{{3 + N_{a} + 2N_{b} }}$	$\frac{{2N\left( {4 - 3D} \right)V_{in} }}{{\left( {1 - D} \right)}}$	$\frac{{\left( {2N\left( {n + 1} \right) + 1} \right)V_{0} }}{{N\left( {n + 1} \right) + 2}}$	$\frac{{2nkV_{0} }}{2 + N + 2nk}$
Diode voltage stress (% of *V*_*0*_)	Min. 6.5 Max. 50	Min. 12.2 Max. 70.8	Min. 15.25 Max. 67	Min 33.25 Max 150	Min 18.95 Max 61
Gain extension technique	Switched inductor	Switched CI	CI, VMCs	CI, built-in transformer	CI, voltage-lift, DCM

### Voltage gain and duty ratio

**Figure 12:**
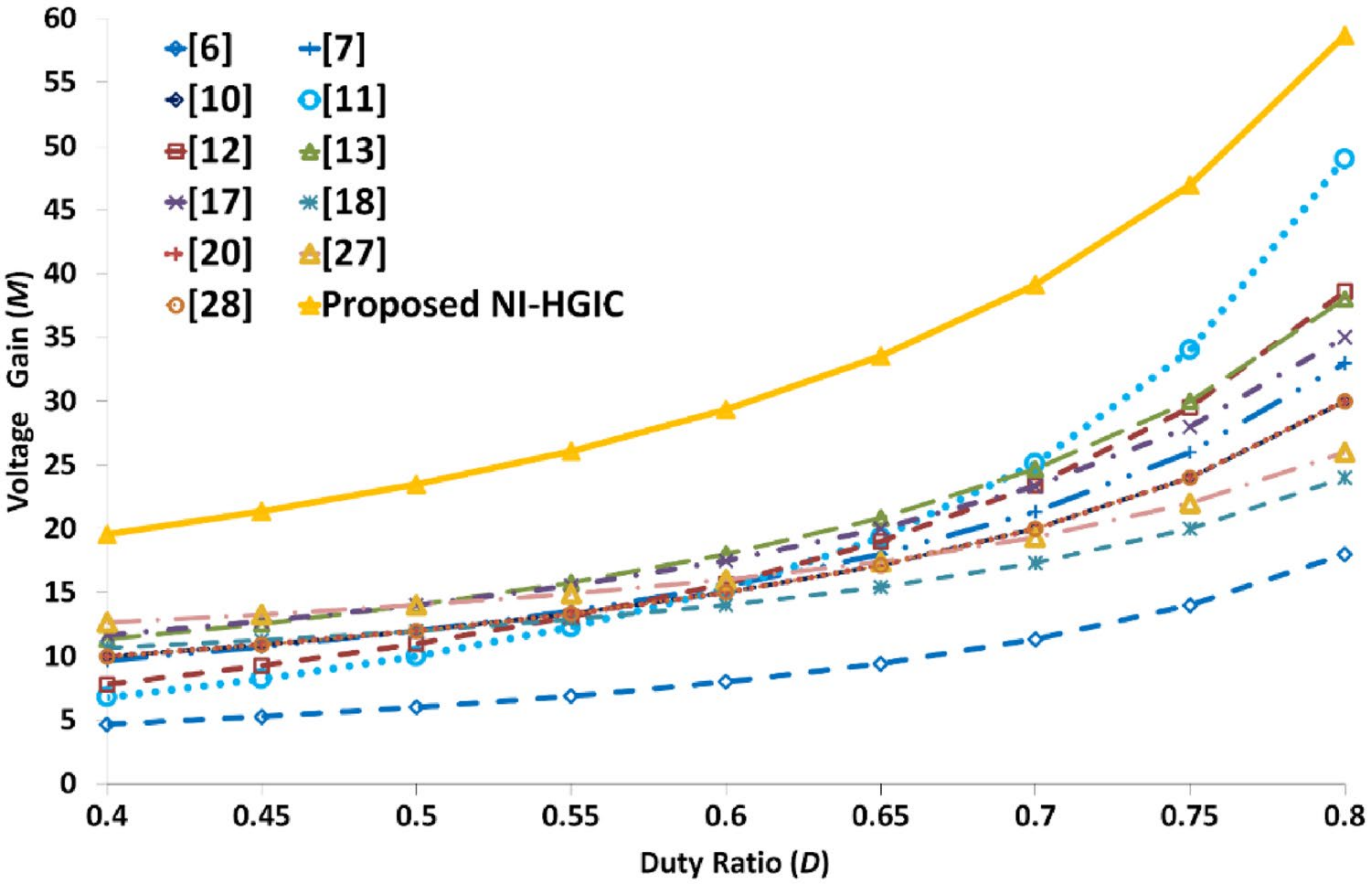
Voltage gain plots of the proposed NI-HGIC and all the converters which are compared.

All the double-switch based converters yield a very high voltage gain value of 15. Among these converters, the converter presented in^10^ operates with the highest voltage gain of 21.5 while the proposed NI-HGIC yields the second highest voltage gain value of 21.11. However, the converter in^10^ operates at a very high duty ratio value of 0.75 while the NI-HGIC operates at a moderate and safe duty ratio value of 0.5. Moreover, the converter in^10^ provides only 237 V at the output which is not a standard DC voltage level. The converter discussed in^6^ operates at the highest duty ratio of 0.78 to provide a voltage gain of 15.2. The duty ratio values of the other two converters presented in ^27,28^ are 0.6 and 0.61 respectively. Despite operating at slightly higher duty ratio values, the voltage gain value of the converters in^27,28^ is only 16 and 15 respectively. In the proposed NI-HGIC, the adopted hybrid gain extension mechanism provides the very high voltage gain value at a safe duty ratio value of 0.5. The main advantages of operating at D = 0.5 are (i) wider range of control to regulate the output voltage especially when input voltage falls steeply, (ii) cancelling the ripple currents through the individual inductors to provide a smooth and ripple-free input current and (iii) reduced conduction losses across the switches and diodes. In fact, the line voltage regulation characteristics and the efficiency value of the proposed NI-HGIC clearly validates the above-mentioned advantage. Figure 12 clearly portrays the high voltage gain capability of the proposed NI-HGIC when compared to both the single and dual switch-based high gain converters.

### Voltage stress on the switches

In all the double-switch converters which are compared in Table 3, the voltage stress magnitude is lower only. In fact, the converters are carefully chosen to appreciate the superior features of the proposed NI-HGIC. The switches of the proposed converter experience the lowest voltage stress which is just 10.5% of *V*_*0*_. The adopted synthesis methodology ensures that the two switches are employed in each phase of the proposed NI-HGIC and is closer to the input port. Resultantly, the switches are subjected to a voltage stress like the single switch in a CBC. Among the compared double-switch converters, the switches of^6^ experience the highest voltage stress magnitudes of 21% and 28.9% of *V*_*0*_. The voltage stress of the other converters is less than 20%. Thus, the advantage of the adopted gain extension mechanism is well-understood.

$$S[12] = \frac{{\left( {N + 3} \right) + \left( {1 + N} \right)K - 2D}}{{nD\left( {N - 2D + K + 3} \right) + ND\left( {5K - nK + 1} \right) + D\left( {K - 1} \right) + 2}} \times DV_{0}$$

### Voltage stress on diodes

The proposed NI-HGIC employs six diodes. Since *D*_*4*_ and *D*_*02*_ are located at the secondary side of the CIs, they are subjected to the maximum voltage stress magnitude which is about 61% of *V*_*0*_. All the other diodes in the proposed NI-HGIC are subjected to reduced voltage stress levels in the range of 18.95% to 37% of *V*_*0*_. The location of the diodes due to the gain extension technique employed in the NI-HGIC is responsible for the relatively less voltage stress magnitude of the diodes. The diodes employed in^28^ experiences the highest voltage stress among all the converters compared. Out of the four diodes used in^28^, two diodes are subjected to voltage stress which is about one-third of *V*_*0*_. The remaining two diodes are located near the output side of the converter and they experience the maximum voltage stress magnitude of 150% of *V*_*0*_. Though converter^28^ employs lesser number of diodes, their voltage stress magnitudes is the highest mainly due to the adopted gain extension technique. Most of the diodes (3 diodes) in^6^ experience voltage stress levels closer to half of *V*_*0*_ while the remaining diodes experience lesser voltage stress. The minimum and maximum values of voltage stress on diodes employed in the converter presented in^11^ are 12.5% and 70.8% *V*_*0*_ respectively. In^11^, half the number of diodes experience higher voltage stress levels (> 50% of *V*_*0*_*)* while the remaining diodes experience lesser voltage stress (< 50% of *V*_*0*_). Three diodes used in^27^ experience a minimum voltage stress magnitude which is 15.25% of *V*_*0*_. Since the remaining two diodes are connected at the secondary of the CIs, their voltage stress is relatively higher at about 67% of *V*_*0*_. To summarize, the adopted gain extension technique which determines the location of the diodes in the power converter circuit impacts the voltage stress undergone by the diodes.

In the proposed NI-HGIC, most of the diodes experience only a lower voltage stress. Hence, while implementing and testing the hardware prototype version, diodes with lower ON-state voltage drop values and low voltage ratings are chosen to enhance the operating efficiency of the NI-HGIC.

### Total component count (*TCC*) and *M/TCC*

To obtain a fair estimate on the components used, the ratio of voltage gain (*M*) to total component count (*TCC*) is calculated and tabulated. All the converters compared in Table 3 yields a very good *M/TCC* value of more than 1; all the converters employ the components judiciously to achieve reasonably higher voltage gain values. Among the two-switch converters, the proposed NI-HGIC uses the maximum number of components; its *TCC* value is the highest. Nevertheless, since it offers the second highest voltage gain at moderate duty ratio value, its *M/TCC* ratio is 1.32. The converter described in^10^ possesses the highest voltage gain value of 21.5 using the second least number of components (*TCC* = 12). Therefore, its *M/TCC* value is also the highest at 1.8. However, as mentioned earlier, the converter in^10^ operates at a duty ratio of D = 0.75 which is rarely preferred. Similar inferences are equally applicable for the converter elaborated in^6,28^ both of which employ 11 components each and operate at higher duty ratio values of 0.78 and 0.61 respectively. The converter in^27^ employs 13 components to achieve a voltage gain value of 16 when its switches are operated at *D* = 0.6. Its *M/TCC* value is the lowest at 1.2.

### Input current ripple

**Figure 13:**
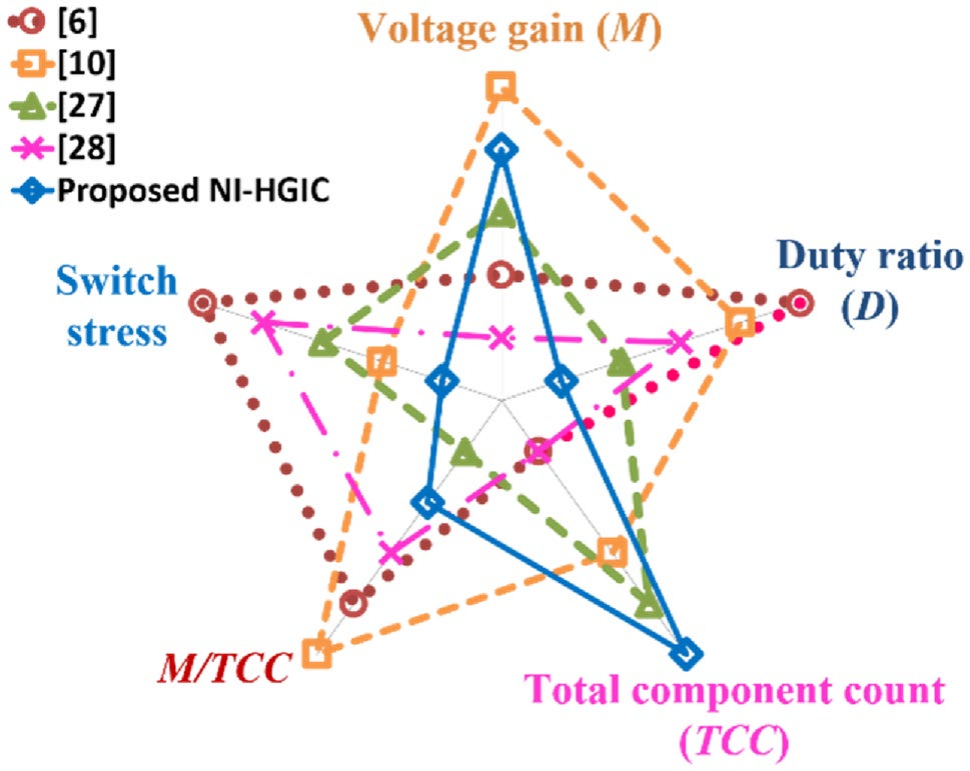
Radial chart demonstrating the beneficial features of the proposed NI-HGIC and other high gain converters.

The converters compared in Table 3 are intended for renewable energy applications like integrating the low voltage PV sources to a high voltage DC bus. To easily implement of maximum power point tracking (MPPT) algorithms, smooth and ripple-free input current is preferred. Hence, the input current ripple is considered as one of the key attributes to estimate the converters’ performance. Three of the five converters (^27,28^ and NI-HGIC) which are compared employ an interleaved structure. However, only the proposed NI-HGIC and the one in^27^ operate with the least input current ripple which is about 11% of the total input current. In the proposed NI-HGIC, the switches are operated at *D* = 0.5 with 180° phase-shift. Resultantly, the individual inductor current ripples get cancelled at the input side. Nevertheless, due to the switching instants and the manufacturing imperfections, the individual inductor currents experience slight glitchy behaviour. Consequently, the input current ripple is about 11% of the total input current value. The converter presented in^6^ employs switched inductor concept and the switches are operated at a very high value which results in the highest input current ripple value of 47%. Though the converter described in^10^ employs a two switched CIs, its input current ripple value is slightly higher at 21% of *I*_*in*_ due to a high duty ratio value. Despite adopting an interleaved structure, the converter elaborated in^28^ draws an input current with 18.75% ripple due to the higher duty ratio value. The radial chart in Fig. 13 summarizes the beneficial features of the proposed NI-HGIC and other similar state-of-the art converters which are compared.

## Conclusion

In this paper, a non-isolated high gain interleaved DC–DC converter was presented. The proposed NI-HGIC was synthesized from a basic IBC structure by initially employing CIs in lieu of discrete inductors. Later, the voltage gain was enhanced by using a voltage-lift capacitor, DCM cells at the primary and secondary side of the CIs. The turns ratio of the CIs was also designed suitably to obtain a practical voltage gain value of 21.11. The prototype NI-HGIC provided an output voltage of 380 V when operated from 18 V input supply and delivered 185W to the load at an efficiency of 94.8% under laboratory test conditions. Due to the judicious synthesis mechanism, the two switches and many of the diodes employed in the NI-HGIC were subjected to a very minimal voltage stress of just 10.5% of the output voltage. The output diode alone was subjected to a higher voltage stress of 61% of *V*_*0*_. Further, as the switches were operated at a duty ratio of 0.5 with 180° phase-shift, the NI-HGIC drew continuous and ripple-free current from the source. The input current ripple was 11.11% mainly due to the leakage effects and mismatch of the custom-made CIs. For verifying the dynamic response, a digital PI controller was implemented and the converter was operated under closed-loop condition. The converter was subjected to line voltage and load current variations. The proposed NI-HGIC responded to dynamic variations swiftly and the output voltage was restored to the nominal operating value. A detailed and fair benchmarking process was carried out by selecting many state-of-the art converters which are available in literature and comparing them with the proposed NI-HGIC. The converters were compared based on several key performance attributes. The comparison proves the superior features of the proposed converter. Some of the salient features of the NI-HGIC are its ability to (i) yield a voltage gain of 21.11 at a safe duty ratio of 0.5, (ii) provide a high voltage conversion value with low voltage stress on the switches and diodes, (iii) draw smooth and ripple-free source current and (iv) quicky respond to dynamic variations in line voltage and load current. The proposed NI-HGIC, when implemented with appropriate protection mechanisms, is expected to be a good candidate topology for DC microgrid application.

## References

[1] Haegel NM, Kurtz RSR (2021). Global progress toward renewable electricity: Tracking the role of solar. IEEE J. Photovolt..

[2] Elavarasan RM (2020). A comprehensive review on renewable energy development, challenges, and policies of leading Indian states with an international perspective. IEEE Access.

[3] Mansour AS, Zaky MS (2023). A new extended single-switch high gain DC–DC boost converter for renewable energy applications. Sci. Rep..

[4] Revathi S, Prabhakar M (2022). Solar PV fed DC microgrid: Applications, converter selection, design and testing. IEEE Access.

[5] Mansour AS, Amer AHH, El-Kholy EE (2022). High gain DC/DC converter with continuous input current for renewable energy applications. Sci. Rep..

[6] de Alcântara Bastos GH, Costa LF, Tofoli FL, Torrico Bascopé GV, Torrico Bascopé RP (2020). Nonisolated DC–DC converters with wide conversion range for high-power applications. IEEE J. Emerg. Select. Top. Power Electron..

[7] Liu, L., & Li, D., Novel modified high step-up DC/DC converters with reduced switch voltage stress. *Int. Trans. Electr. Energy Syst.*, 1–24 (2022).

[8] Hoseinzadeh Lish M, Ebrahimi R, Madadi Kojabadi H, Guerrero JM, Nourani Esfetanaj N, Chang L (2020). Novel high gain DC–DC converter based on coupled inductor and diode capacitor techniques with leakage inductance effects. IET Power Electron..

[9] Afzal R, Tang Y, Tong H, Guo Y (2020). A high step-up integrated coupled inductor-capacitor DC–DC converter. IEEE Access.

[10] Mirzaee A, Moghani JS (2020). Coupled inductor-based high voltage gain DC–DC converter for renewable energy applications. IEEE Trans. Power Electron..

[11] Ebrahimi R, Madadi Kojabadi H, Chang L, Blaabjerg F (2019). Coupled-inductor-based high step-up DC–DC converter. IET Power Electron..

[12] Tarzamni H, Kolahian P, Sabahi M (2021). High step-up DC–DC converter with efficient inductive utilization. IEEE Trans. Industr. Electron..

[13] Fan X, Sun H, Yuan Z, Li Z, Shi R, Ghadimi N (2020). High voltage gain DC/DC converter using coupled inductor and VM techniques. IEEE Access.

[14] Chen M, Yin C, Loh PC, Ioinovici A (2020). Improved large DC gain converters with low voltage stress switches based on coupled-inductor and voltage multiplier for renewable energy applications. IEEE J. Emerg. Select. Top. Power Electron..

[15] Schmitz DCM, Coelho RF (2020). Comprehensive conception of high step-up DC–DC Converters with coupled inductor and voltage multipliers techniques. IEEE Trans. Circuits Syst I Regul. Pap..

[16] Yu D, Yang J, Xu R, Xia Z, Iu HH-C, Fernando T (2018). A family of module-integrated high step-up converters with dual coupled inductors. IEEE Access.

[17] Forouzesh M, Shen Y, Yari K, Siwakoti YP, Blaabjerg F (2018). High-efficiency high step-up DC–DC converter with dual coupled inductors for grid-connected photovoltaic systems. IEEE Trans. Power Electron..

[18] Eskandarpour Azizkandi M, Sedaghati F, Babaei E (2020). A topology of coupled inductor DC–DC converter with large conversion ratio and reduced voltage stress on semiconductors. IET Power Electron..

[19] Hosseinzadehlish M, Hashemzadeh SM, Pourjafar S, Babaei E (2022). A single switch high step-up DC–DC converter based on tri-winding coupled inductor for renewable energy applications. Int. Trans. Electr. Energy Syst..

[20] Azizkandi ME, Sedaghati F, Shayeghi H, Blaabjerg F (2020). A high voltage gain DC–DC converter based on three winding coupled inductor and voltage multiplier cell. IEEE Trans. Power Electron..

[21] Wu G, Ruan X, Ye Z (2018). High step-up DC–DC converter based on switched capacitor and coupled inductor. IEEE Trans. Industr. Electron..

[22] Alghaythi M, O'connell R, Islam N, Khan M, Guerrero J (2020). A high step-up interleaved DC–DC converter with voltage multiplier and coupled inductors for renewable energy systems. IEEE Access.

[23] Moradisizkoohi H, Elsayad N, Mohammed OA (2020). An integrated interleaved ultrahigh step-up DC–DC converter using dual cross-coupled inductors with built-in input current balancing for electric vehicles. IEEE J. Emerg. Select. Top. Power Electron..

[24] Samuel VJ, Keerthi G, Prabhakar M (2020). Ultra-high gain DC–DC converter based on interleaved quadratic boost converter with ripple-free input current. Int. Trans. Electr. Energy Syst..

[25] Samuel VJ, Keerthi G, Mahalingam P (2020). Interleaved quadratic boost DC–DC converter with high voltage gain capability. Electr. Eng..

[26] Samuel VJ, Keerthi G, Mahalingam P (2020). Coupled inductor-based DC–DC converter with high voltage conversion ratio and smooth input current. IET Power Electron..

[27] Alzahrani MF, Shamsi P (2019). A family of scalable non-isolated interleaved DC–DC boost converters with voltage multiplier cells. IEEE Access.

[28] Hashemzadeh, S.M., Babaei, E., Hosseini, S.H., & Sabahi, M, Design and analysis of a new coupled inductor based interleaved high step-up DC–DC converter for renewable energy applications. *Int. Trans. Electr. Energy Syst.*, 1–14 (2022).

[29] Mohammadi Jouzdani, Marzieh & Shaneh, Mahdi & Nouri, Tohid, Design of an interleaved high step-up DC–DC converter with multiple magnetic devices for renewable energy systems applications. *Int. Trans. Electr. Energy Syst.*, 1–14 (2022).

[30] Seo S-W, Lim D-K, Choi HH (2020). High step-up interleaved converter mixed with magnetic coupling and voltage lift. IEEE Access.

[31] Samuel VJ, Keerthi G, Mahalingam P (2020). Non-isolated DC–DC converter with cubic voltage gain and ripple-free input current. IET Power Electron..

[32] Ahmed NA, Alajmi BN, Abdelsalam I, Marei MI (2022). Soft switching multiphase interleaved boost converter with high voltage gain for EV applications. IEEE Access.

